# Knowledge, attitude, practices and treatment-seeking behaviour concerning cutaneous leishmaniasis among rural hyperendemic communities in western Yemen

**DOI:** 10.1038/s41598-024-63526-6

**Published:** 2024-06-03

**Authors:** Manal A. Al-Ashwal, Abdulelah H. Al-Adhroey, Wahib M. Atroosh, Sheikh Abdulhafed Alshoteri, Assia Abdullah Al-Subbary, Talal H. Alharazi, Hany Sady, Meram Azzani, Yee-Ling Lau, Hesham M. Al-Mekhlafi

**Affiliations:** 1 Department of Parasitology, Faculty of Medicine, Universiti Malaya, 50603 Kuala Lumpur, Malaysia; 2 Department of Community Medicine, Faculty of Medicine, Thamar University, Dhamar, Yemen; 3 Department of Microbiology and Parasitology, Faculty of Medicine and Health Sciences, University of Aden, Aden, Yemen; 4 National Centre of Public Health Laboratories, Ministry of Health and Population, Dhamar, Yemen; 5 Department of Clinical Laboratory Sciences, College of Applied Medical Sciences, University of Hail, Hail, Kingdom of Saudi Arabia; 6 Department of Medical Parasitology, Faculty of Medicine and Health Sciences, Taiz University, Taiz, Yemen; 7 Centre for Child, Adolescent and Maternal Health Research, Faculty of Medicine and Health Technology, Tampere University, Tampere, Finland; 8 Department of Public Health Medicine, Faculty of Medicine, Universiti Teknologi MARA, 47000 Sungai Buloh, Selangor, Malaysia; 9 Department of Epidemiology, Faculty of Public Health and Tropical Medicine, Jazan University, 45142 Jazan, Saudi Arabia; 10 Department of Parasitology, Faculty of Medicine and Health Sciences, Sana’a University, Sana’a, Yemen

**Keywords:** Leishmaniasis, Infectious diseases, Neglected tropical diseases, KAP survey, Yemen, Parasitic infection, Risk factors

## Abstract

Cutaneous leishmaniasis (CL), a neglected tropical disease (NTD), is a major public health problem in Yemen with widespread distribution in rural areas. Evaluating the knowledge and understanding of people’s beliefs towards the disease is essential to the implementation of effective control measures. This study aims to assess the knowledge, attitudes, practices (KAP) and treatment-seeking behaviour concerning CL among rural populations in the western highlands of Yemen. A community-based cross-sectional study was conducted among 289 household heads in four rural areas of the Utmah District. Data were collected using a pre-tested questionnaire. All the participants had heard about the disease; however, only 9.3% attributed it to sandflies. Nearly half (48.1%) of the participants could not mention any preventive measures for CL, and nearly two-thirds (65.4%) could not do so for sandflies. The overall ‘good’ knowledge about CL was found to be 51.2%, and it was 33.9% for sandflies. The participants’ attitude and prevention practices towards CL were not satisfactory, as only 38.1% and 16.3% had a positive attitude and good CL-related prevention practices, respectively. Moreover, 45.7% believed CL to be a stigmatising disease, and 50% had used traditional remedies to treat suspected CL lesions. Multivariate analyses showed that age, sex, presence of CL-confirmed cases in the same household, residency, occupation and monthly household income were the significant predictors associated with KAP concerning CL among the participants. The findings support an urgent need for integrated health education and community mobilisation interventions to improve awareness of these vulnerable populations about this devastating disease.

## Introduction

Cutaneous leishmaniasis, a neglected tropical disease (NTD), is a disfiguring vector-borne disease caused by protozoan parasites of the genus *Leishmania* and transmitted by the bites of infected female sandflies^1^. It is estimated that about 0.7 to 1.2 million new cases of CL occur annually worldwide^2,3^, and about 40 million people worldwide are currently living with inactive CL scarring^4^. However, it is widely believed that the true incidence and burden of CL are grossly underestimated^3^. The disease is endemic in more than 90 countries; however, about 80% of the cases reported worldwide occur in the Middle East and North Africa (MENA) region (mainly Algeria, Iran, Syria, Afghanistan, Pakistan, Iraq, Yemen and Saudi Arabia)^2,3,5–7^.

The clinical manifestation starts with a skin lesion at the vector bite site (usually without pain or pruritus) and progressively increases in size to form a rounded nodule that is usually exposed to secondary bacterial or fungal infections. It can then produce purulent and painful ulcers, mainly on the exposed parts of the body, leaving life-long scars and serious disability or severe social stigma^1,8^. Several risk factors for CL have been identified. These include but are not limited to poverty, young age, climate change, specific occupations and activities (e.g. farming, military, mining and hunting), migration, deforestation, malnutrition, illiteracy and lack of preventive measures^1,9–12^. Adherence to treatment and preventive measures is crucial to control CL; however, adherence is influenced by people’s knowledge and attitude toward the disease and its vector^13^. Knowledge, attitude, and practices (KAP) surveys are frequently employed tools for collecting essential data to guide the development of CL control and prevention strategies and interventions. Several KAP surveys on CL have been conducted in many countries across the MENA region such as Pakistan^14^, Iran^15^, Saudi Arabia^16^, and Morocco^17^, as well as other countries in East Africa^18^ and Southeast Asia^19^.

In Yemen, CL is a public health problem particularly in rural areas^20^. CL is the most common form of leishmaniasis, with more than 12,000 cases reported in 2019^21,22^. Several previous studies have revealed the occurrence of CL in all regions across Yemen, with the highest rates reported in the Dhamar, Al-Bayda and Hajjah governorates^20,23–26^. However, there is a dearth of studies evaluating the population awareness towards CL, with only one study conducted in Taiz Governorate, southwestern Yemen (about 160 km to the south of Dhamar)^27^. Therefore, the present study aims to assess the knowledge, attitudes, practices (KAP) and treatment-seeking behaviour regarding CL, and the knowledge about the sandfly vector among the rural population of CL-hyperendemic areas in the Utmah District, western highlands of Yemen.

## Methods

### Ethical statement

Ethical approval for this study was obtained from the Medical Ethics Committee of the Universiti Malaya Medical Centre, Kuala Lumpur, Malaysia (Ref. No. 201411–805), and also from the Medical Ethics Committee of Thamar University, Yemen (Ref. No. TUMEC-17018). Before the commencement of data collection, the participants were informed about the study objectives and procedures. Subsequently, a written and signed or thumb printed informed consent was obtained from each participant. The study was performed in accordance with relevant guidelines and regulations. All procedures were performed in accordance with the ethical standards laid down in the 1964 Declaration of Helsinki and its later amendments.

### Study design

A community-based cross-sectional survey was carried out between January 2017 and May 2019 among the rural population of the Utmah District in the western highlands of Yemen. The main study involved three components: questionnaire surveys, physical examinations and parasitological examinations^28^. Household heads were interviewed to collect information on their KAP concerning CL and its sandfly vector.

### Study area

Utmah district, one of the 12 districts of Dhamar Governorate, is located between longitude 43.95°E and latitude 14.66°N. The district occupies a total land area of about 460 km^2^ and has a total population of about 145,000. It consists of five sub-districts, called “*Mekhlaf*” that comprise 57 areas, called “*Uzlat*”. Utmah district is a highland area representing the natural extension of the Sarawat mountain range, at an elevation of 920–2800 m above sea level^29^. The climate varies between wet and arid, with warm summers and cold winters. The district has a mean annual temperature of 22°C and a mean annual rainfall of 750–800 mm.

**Figure 1:**
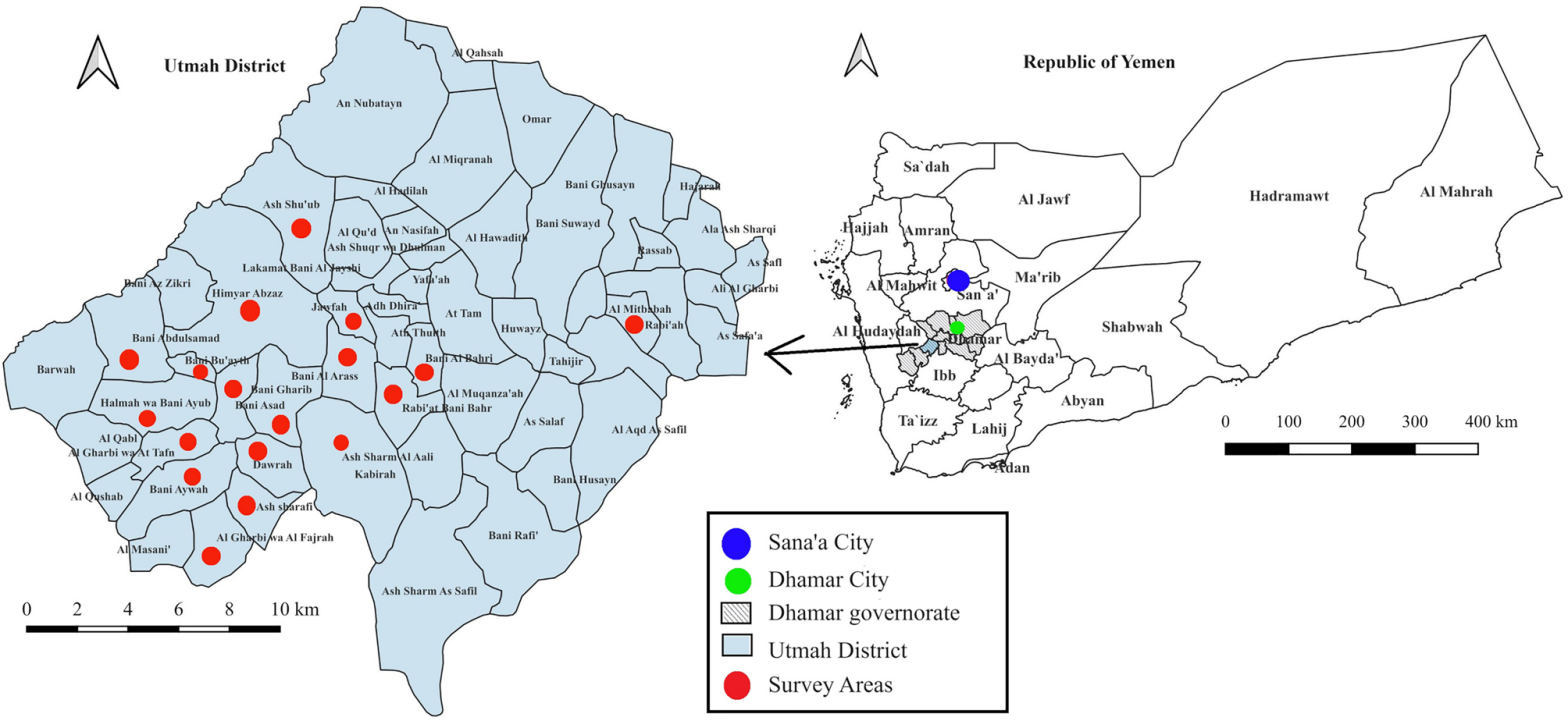
A map of the study area in Utmah district, western Yemen.

Eighteen areas representing four sub-districts (Alsomal, Razeh, Hemiar Alwasat and Bani Bahr) were selected for this study (Fig. 1). The selection was based on the following criteria: (1) high incidence of CL, (2) accessibility and (3) security. Due to armed conflicts during the study period, Samah, the fifth sub-district with 10 areas, was not included^30^. More information on the study areas and selection of households has been published elsewhere^28^.

### Study population and sample size

In Yemen, the detection of leishmaniasis cases relies solely on passive case detection, and there are no national control programmes for leishmaniasis and its sandfly vector. However, the Regional Leishmaniasis Control Centre (RLCC), a charitable non-governmental organisation founded in early 2013, has made invaluable efforts through treatment and awareness campaigns targeting leishmaniasis in certain areas in Yemen where CL is endemic^31^. Unfortunately, these efforts have been largely affected by a lack of resources and the ongoing civil war that started in 2015^31^.

The sample size was estimated based on the WHO’s practical manual for sample size determination in health studies^32^. At a confidence interval of 95% and a desired margin of 5% and based on the percentage of respondents with good CL-related knowledge (22.3%)^27^, the minimum sample size required for this study was 267. An additional 20% was added to account for possible non-response, resulting in a total sample size of 320. Overall, out of 320 households, 289 (90.3%) were included in the study, with 31 household heads refusing to participate.

In each area, the selection of households was done by simple random sampling using a list of households and maps compiled by the districts’ administrative or health officials. In these areas, the houses are dispersed or scattered over the mountainous terrain without any clear pattern; nonetheless, every second household was selected for the study. If the residents of the selected house were unavailable or if the head of the household declined to allow his/her house to take part in the study, the household was not involved in the survey. In this case, the next household was approached; if involved, then the selection of every second household continued until the sample size was achieved. Both male and female adults who had been in the study area for one year and gave their agreement to voluntarily take part in the study were considered eligible for inclusion in the study. To ensure that the participants were the main decision-making individuals within the household, the study participants for this KAP survey were preferably the household heads if available during the time of the home visit; otherwise, wives of heads of households were invited to participate in the survey.

### Questionnaire survey

A pre-tested questionnaire was used for data collection. The questionnaire was prepared in English and then translated into the participants’ native language (Arabic). To assess its feasibility and validity, the questionnaire was pre-tested, in the targeted district, on 30 household heads from two villages that were not involved in the survey but had similar characteristics. The questionnaire was designed to collect information on participants’ demographics, socioeconomic background, housing conditions, and environmental conditions. Also, the questionnaire involved questions about the participants’ knowledge about the disease and its sandfly vector. In addition, the questionnaire also included questions to assess participants’ attitude towards CL and their CL-related practices. The participants were interviewed face-to-face in their household settings by two research assistants who received appropriate training on questionnaire administration for this survey.

### Data analysis

Data were reviewed and double checked before and after data entry. The data were analysed using the *IBM SPSS Statistics* (version 20). For descriptive data, frequency, percentage, and mean (standard deviation) or median (interquartile range) were used, where applicable, to describe the KAP components and independent variables. Scores were developed for the participants’ responses on questionnaire items related to knowledge, attitude and practices following the methods described previously^18,27^. In brief, from the KAP questionnaire, a composite score of each component was calculated for each participant where each correct response received a score of 1 and each incorrect or unsure response received a score of 0. The total scores were further dichotomized based on the overall scores of each component. The total knowledge scores ranged from 0 to 5, and scores between 0 and 3 were categorised as poor knowledge, while scores between 4 and 5 were considered indicative of good knowledge. Similarly, total attitude scores between 0 and 3 were classified as negative attitude, while scores between 4 and 5 indicated positive attitude. With regards to prevention practices, the total scores ranged from 0 to 10. Scores between 0 and 5 were categorised as poor practices, while scores between 6 and 10 reflected good practices.

Associations between participants’ KAP variables and socio-demographic factors including age, sex, residence, education level, occupation, monthly household income (a poverty income threshold of YER50,000 equivalent to US$75 was estimated based on the 2014 Household Budget Survey data)^33^, and the presence of confirmed CL cases in the household were examined by the Chi-square test or Fisher’s exact test. Multivariate backward logistic regression analyses were performed to identify the significant predictors of good CL- and sandfly-related knowledge, positive attitude towards CL and good CL prevention practices. All variables with a *P* value of ≤ 0.25 in the univariate analyses were included in the logistic regression analyses^34^. Adjusted odds ratios (AORs) with corresponding 95% CIs were computed and the *P* value of less than 0.05 was considered statistically significant.

## Results

### Socio-demographic characteristics of the participants

Two hundred eighty-nine household heads (75.1% male, 24.9% female) from rural areas of the Utmah District participated in this study. The mean age of the participants was 43.2 ± 12.7 years. Overall, 152 (52.6%) of the participants were non-educated, while 47.4% had attained at least six years of formal education (primary education). A total of 130 (45.0%) were mainly involved in farming activities, while 26 (9.0%) were government employees. Moreover, only 80 participants (27.7%) had a total monthly household income of YER50,000 (equivalent to US$75) and above, while 209 participants (72.3%) had a total monthly household income of less than YER50,000. Almost all (94.1%) of the participants used unimproved sources for drinking water, such as rain, wells and springs. There was no proper disposal of waste and garbage. Supplementary Table S1 presents the general characteristics of the participants.

### Knowledge about cutaneous leishmaniasis

Table 1 shows the results of the participants’ knowledge about the signs and symptoms, transmission, prevention and treatment of CL. It was found that all the participants had prior knowledge about the disease in its local name. However, only 69.6% (201/289) had heard of the scientific terms ‘*Leishmania’* or ‘leishmaniasis’, and only 4.2% (12/289) correctly identified the disease as a fly-induced skin disease. The participants mentioned some local names to describe the CL lesion, such as *sheqna* (lesion spreads among all family members), *bada’h* or *budda* (popular/nodular lesion at the initial stage), *bulla* (wet lesion) and *akela* (skin-eating, ulcerative skin lesion). Knowledge about the scientific terms was mostly through medical teams who visited the area (52.2%; 105/201), followed by the clinics where they sought treatment (26.4%; 53/201) and 19.9% (40/201) from family members, friends and neighbours. Over half of the participants (51.9%; 150/289) did not know the cause of CL, but 9.3% attributed it to sandflies and 18.6% to mosquitoes (not specifically sandflies).

**Table 1:** Knowledge about cutaneous leishmaniasis among the study participants (n = 289).

Variables	Response	N (%)
Know the disease by the term	Leishmania or leishmaniasis	201 (69.6)
	Local names only	76 (26.2)
	Fly-induced skin disease	12 (4.2)
Source of information about the term leishmaniasis (n = 201)	Medical team visits	105 (52.2)
	Clinic	53 (26.4)
	Family, friends & neighbours	40 (19.9)
	School	3 (1.5)
Knowledge about causes and transmission of CL	Bites of mosquitoes/insects	55 (18.6)
	Bites of sandflies	27 (9.3)
	Houseflies	13 (4.5)
	Through animals	25 (8.7)
	Human-to-human	20 (6.9)
	Do not know	150 (51.9)
Knowledge about symptoms of CL	Skin lesion	149 (55.0)
	Skin wound	52 (18.0)
	Skin scar	46 (15.9)
	Itching and redness	32 (11.1)
Knowledge about prevention methods of CL	Treating patients	41 (14.2)
	Using insecticide	33 (11.4)
	Improved personal hygiene	29 (10.0)
	Isolating patients	26 (9.0)
	Using bed nets	21 (7.3)
	Do not know	139 (48.1)
Knowledge about treatment options of CL	Chemotherapy	83 (28.7)
	Herbal medicine	59 (20.4)
	Cauterisation	34 (11.8)
	No treatment	16 (5.5)
	Self-heal	10 (3.5)
	Do not know	87 (30.1)
Overall knowledge about CL		
Total mean score ± SD	–	3.3 ± 1.2
Good knowledge (score 4–5)	–	148 (51.2)
Poor knowledge (score 0–3)	–	141 (48.8)

When participants were asked about the prevention of CL, 48.1% (139/289) could not cite any preventive measure, while 11.4% mentioned using insecticides, 14.2% mentioned treating infected patients and 7.3% mentioned using bed nets (Table 1). Based on the scoring system, 51.2% of the participants had a good level of knowledge about CL while 48.8% had a poor level.

### Knowledge about the sandfly vector

Although all the participants had prior knowledge about the sandfly in its local name, only 26.6% (77/289) of them had heard the term ‘sandfly’ in its Arabic translation (Table 2). Moreover, 36.0% (104/289) were able to describe and differentiate sandflies from other common mosquitoes and insects based on some correct feature of the vector.

**Table 2:** Knowledge about the sandfly vector among the study participants (n = 289).

Variables	Response categories	N (%)
Know the sandfly vector by the term	Sandfly	77 (26.6)
	Only local name	212 (73.4)
Source of information about the term sandfly (n = 77)	Medical team visits	20 (26.0)
	Clinic	5 (6.5)
	Family, friends & neighbours	52 (67.5)
Can identify and differentiate sandflies from other mosquitoes and common flies	Yes	104 (36.0)
	No	185 (64.0)
Know breeding places of sandflies	Cattle barns	49 (17.0)
	Valleys	24 (8.3)
	Old houses	22 (7.6)
	Caves	16 (5.5)
	Wells	11 (3.8)
	Deserts	9 (3.1)
	Do not know	158 (54.7)
Know biting time of sandflies	During night time	124 (42.9)
	During day time	21 (7.3)
	At any time	51 (17.6)
	Do not know	93 (32.2)
Know methods to control sandflies	Spraying insecticides	53 (18.3)
	Screening of windows	14 (4.8)
	Cleanliness	13 (4.5)
	Using bed nets	11 (3.8)
	Using mosquito coil smoke	9 (3.1)
	Do not know	189 (65.4)
Overall knowledge about sandflies		
Total mean score ± SD	–	2.6 ± 1.3
Good knowledge (score 4–5)	–	98 (33.9)
Poor knowledge (score 0–3)	–	191 (66.1)

Regarding breeding places of sandflies, a majority (54.7%; 158/289) of the participants did not know the answer, while 17.0%, 8.3% and 5.5% mentioned domestic animal barns, valleys and caves, respectively. Moreover, 65.4% (189/289) of the participants were unable to mention any control measures against sandflies. Overall, only 33.9% of the participants had a good or satisfactory level of knowledge about sandflies, while about two-thirds (66.1%) had a poor level.

### Attitudes and prevention practices concerning cutaneous leishmaniasis

Table 3 shows that a total of 246 participants (85.1%) considered CL to be a serious disease. The majority (76.5%) believed that CL is a curable disease, while 9.0% believed that the disease cannot be cured. Only 27.3% of the participants believed that CL can be prevented. In addition, 45.7% (132/289) believed that CL is a stigmatising disease, with a significantly higher percentage of them being female compared to male (56.9% vs. 41.9%; χ^2^ = 4.908; *P* = 0.027). Overall, it was found that only 38.1% (110/289) of the participants had an overall positive or favourable attitude towards CL.

**Table 3:** Attitude towards CL among the study participants (n = 289).

Variables	Response categories	N (%)
Is CL a serious disease?	Yes*	246 (85.1)
	No	28 (9.7)
	Do not know	15 (5.2)
Is CL curable?	Yes*	221 (76.5)
	No	26 (9.0)
	Do not know	42 (14.5)
Is CL preventable?	Yes*	79 (27.3)
	No	167 (57.8)
	Do not know	43 (14.9)
I believed that having close contact with individuals infected with CL is safe	Yes*	175 (60.6)
	No	60 (20.8)
	Do not know	54 (18.7)
Is CL a stigmatising disease?	Yes	132 (45.7)
	No*	157 (54.3)
Overall attitude status		
Total mean score ± SD	–	3.1 ± 1.2
Positive attitude (score 4–5)	–	110 (38.1)
Negative attitude (score 0–3)	–	179 (61.9)

*Correct responses were assigned a score of 1 and other responses were assigned 0.

Table 4 shows the results of the participants’ CL-related prevention practices. Widespread poor practices were observed among the participants, with only 16.3% (47/289) of them having good CL-related prevention practices in general. The results revealed that only 9% (26/289) of the participants used bed nets and 9.3% (27/289) used insecticides, and 13.8% (40/289) had screened windows with nets. Similarly, only 18.3% (53/289) of the participants lived in good houses (built of brick or stone with no cracks in the walls).

**Table 4:** Participants’ prevention practices against CL in Utmah district, western Yemen.

Variables	Response categories	N (%)
Using bed nets	Yes*	26 (9.0)
	No	263 (91.0)
Using insecticide spray in the household	Yes*	27 (9.3)
	No	262 (90.7)
Living in a good house (no cracks in walls)	Yes*	53 (18.3)
	No	236 (81.7)
Living near open water sources (≤ 250 m)	Yes	179 (61.9)
	No*	110 (38.1)
Working outside during night	Yes	103 (35.6)
	No*	186 (64.4)
Sleeping outside during night	Yes	88 (30.4)
	No*	201 (69.6)
Wearing long sleeve clothes	Yes*	149 (51.6)
	No	140 (48.4)
Keeping windows closed during night	Yes*	87 (30.1)
	No	202 (69.9)
Screening of windows with nets	Yes*	40 (13.8)
	No	249 (86.2)
Keeping livestock animals outside the house	Yes*	68 (23.5)
	No	221 (76.5)
Overall practices level		
Total mean score ± SD	–	3.9 ± 1.7
Good (score 6–10)	–	47 (16.3)
Poor (score 0–5)	–	242 (83.7)

*Correct responses were assigned a score of 1 and other responses were assigned 0.

### Treatment-seeking behaviour concerning cutaneous leishmaniasis

Table 5 shows that 56.7% of the participants mentioned that they would go to the nearest health centre when they have skin lesions, while 28.4% would preferably use traditional herbal remedies for the treatment of skin lesions, and 10.4% indicated that they do not treat skin lesions. However, when questioned about how they had previously treated skin lesions (probably due to CL) for themselves or their family members, a substantial number (50%; 79/158) stated they used traditional herbal remedies, followed by seeking treatment from health centres (31.7%).

**Table 5:** Treatment-seeking behaviour and practices of participants in relation to CL in the Utmah district, western Yemen.

Variables	N (%)
Treatment-seeking behaviour of skin lesions (as a first line activity)	
Go to hospital/clinic	164 (56.7)
Use traditional herbal remedies	82 (28.4)
Cauterisation	13 (4.5)
Do nothing	30 (10.4)
Treatment of previous CL-suspected lesions (n = 158)	
Medication from health centres	50 (31.7)
Traditional medicinal plants	79 (50.0)
Cauterisation	13 (8.2)
Application of acids	9 (5.7)
Did nothing	7 (4.4)
Medicinal plants used by the respondents (n = 79) Scientific name (local name/part used)	
*Aloe vera* (Saber/leaf latex & gel)	8 (10.1)
*Calotropis procera* (Oshar/milky latex)	6 (7.6)
*Ficus palmata* (Hamat/milky latex)	5 (6.3)
*Acalypha fruticosa* (Enshit/leaves)	5 (6.3)
Prunus dulcis (Lauz/flowers)	3 (3.8)
Ready herbal remedies from traditional healers	44 (55.7)
Did not remember	8 (10.1)

Interestingly, 8.2% (13/158) used cauterisation of the lesions with very hot metal objects (spoons, knives or iron skewers), and 5.7% (9/158) applying caustic materials or strong acids, such as battery acid on the lesions. Of the 79 participants who applied traditional remedies, 44 (55.7%) used herbal remedies from traditional healers, some medicinal plants were also mentioned, including *Aloe vera, Calotropis procera, Ficus palmate*, *Acalypha fruticosa* and *Prunus dulcis*.

### Factors associated with KAP concerning CL and the sandfly vector

Table 6 shows that monthly household incomes of ≥ YER50,000 was significantly associated with good knowledge about CL (*P* = 0.035). People over the age of 40 years were significantly associated with a good knowledge about the sandfly vector (*P* = 0.014) while living in Bani Bahr sub-district was significantly associated with lower level of knowledge about the vector (*P* = 0.015). Table 7 shows that females (*P* = 0.019), living in Razeh sub-district (*P* = 0.018) and living in a household with a confirmed CL case (*P* = 0.025) were significant factors associated with good attitude towards CL. Moreover, having a secondary and above education level (*P* = 0.031) and monthly household incomes of ≥ YER50,000 (*P* = 0.033) significantly associated with good prevention practices related to CL (Table 7).

**Table 6:** Univariate analysis for association of participants’ knowledge towards CL and sandfly vector with their socio-demographic factors.

Variables	Categories	Knowledge about CL^c^				Knowledge about sandfly^d^			
		Poor N (%)	Good N (%)	COR (95% CI)	*P*	Poor N (%)	Good N (%)	COR (95% CI)	*P*
Age (years)	> 40	77 (52.0)	71 (48.0)	0.77 (0.48, 1.22)	0.259	88 (59.5)	60 (40.5)	1.85 (1.13, 3.04)	0.014^a,b^
	18–40	64 (45.4)	77 (54.6)	1		103 (73.0)	38 (27.0)	1	
Gender	Female	42 (58.3)	30 (41.7)	0.60 (0.35, 1.03)	0.062^b^	44 (61.1)	28 (38.9)	1.34 (0.77, 2.32)	0.303
	Male	99 (45.6)	118 (54.4)	1		147 (67.7)	70 (32.3)	1	
Location	Hemiar Alwasat	8 (33.3)	16 (66.7)	1.78 (0.69, 4.60)	0.232^b^	17 (70.8)	7 (29.2)	0.51 (0.19, 1.36)	0.172^b^
	Bani Bahr	54 (48.6)	57 (51.4)	0.94 (0.53, 1.65)	0.825	80 (72.1)	31 (27.9)	0.48 (0.26, 0.87)	0.015^a,b^
	Razeh	39 (56.5)	30 (43.5)	0.68 (0.36, 1.30)	0.243^b^	47 (68.1)	22 (31.9)	0.58 (0.31, 1.12)	0.105^b^
	Alsomal	40 (47.1)	45 (52.9)	1		47 (55.3)	38 (44.7)	1	
Level of education	Secondary & above	33 (45.2)	40 (54.8)	1.19 (0.67, 2.07)	0.561	50 (68.5)	23 (31.5)	089 (0.49, 1.61)	0.687
	Primary	33 (51.6)	31 (48.4)	0.92 (0.51, 1.64)	0.766	41 (64.1)	23 (35.9)	1.08 (0.59, 2.01)	0.808
	Non educated	75 (49.3)	77 (50.7)	1		100 (65.8)	52 (34.2)	1	
Occupation	Government employees	9 (34.6)	17 (65.4)	1.65 (0.61, 4.48)	0.322	17 (65.4)	9 (34.6)	1.17 (0.42, 3.27)	0.761
	Self-employed workers	36 (40.9)	52 (59.1)	1.26 (0.61, 2.61)	0.526	62 (70.5)	26 (29.5)	0.93 (0.43, 2.03)	0.852
	Farmers	75 (57.7)	55 (42.3)	0.64 (0.33, 1.27)	0.200^b^	81 (62.3)	49 (37.7)	1.34 (0.65, 2.76)	0.428
	Not working	21 (46.7)	24 (53.3)	1		31 (68.9)	14 (31.1)	1	
Monthly household income	≥ 50,000 YER	31 (38.8)	49 (61.2)	1.76 (1.04, 2.97)	0.035^a,b^	49 (61.2)	31 (38.8)	1.34 (0.79, 2.29)	0.282
	< 50,000 YER	110 (52.6)	99 (47.4)	1		142 (67.9)	67 (32.1)	1	
Presence of confirmed CL cases among family members	Yes	20 (37.7)	33 (62.3)	1.74 (0.94, 3.20)	0.075^b^	40 (75.5)	13 (24.5)	0.58 (0.29, 1.14)	0.110^b^
	No	121 (51.3)	115 (48.7)	1		151 (64.0)	85 (36.0)	1	

*COR* crude odds ratio. *CI* confidence interval.

^a^Significant association (*P* < 0.05).

^b^Included in multivariate analyses (*P* ≤ 0.25).

^c^Based on scores shown in Table 1.

^d^Based on scores shown in Table 2.

**Table 7:** Univariate analysis for association of participants’ attitude and prevention practices towards CL with their socio-demographic factors.

Variables	Categories	Attitudes towards CL^c^				Prevention practices against CL^d^			
		Negative N (%)	Positive N (%)	COR (95% CI)	*P*	Poor N (%)	Good N (%)	COR (95% CI)	*P*
Age (years)	> 40	87 (58.8)	61 (41.2)	1.32 (0.82, 2.12)	0.258	123 (83.1)	25 (16.9)	1.10 (0.59, 2.06)	0.767
	18–40	92 (65.2)	49 (34.8)	1		119 (84.4)	22 (15.6)	1	
Gender	Female	53 (73.6)	19 (26.4)	0.50 (0.28, 0.90)	0.019^a,b^	58 (80.6)	14 (19.4)	1.35 (0.67, 2.69)	0.399
	Male	126 (58.1)	91 (41.9)	1		184 (84.8)	33 (15.2)	1	
Location	Hemiar Alwasat	16 (66.7)	8 (33.3)	0.72 (0.28, 1.85)	0.488	19 (79.2)	5 (20.8)	1.23 (0.40, 3.81)	0.911
	Bani Bahr	60 (54.1)	51 (45.9)	1.21 (0.69, 2.15)	0.505	94 (84.7)	17 (15.3)	0.84 (0.39, 1.81)	0.662
	Razeh	53 (76.8)	16 (23.2)	0.43 (0.21, 0.87)	0.018^a,b^	59 (85.5)	10 (14.5)	0.79 (0.33, 1.89)	0.779
	Alsomal	50 (58.8)	35 (41.2)	1		70 (82.4)	15 (17.6)	1	
Level of education	Secondary & above	41 (56.2)	32 (43.8)	1.30 (0.74, 2.29)	0.363	55 (75.3)	18 (24.7)	2.16 (1.06, 4.39)	0.031^a,b^
	Primary	43 (67.2)	21 (32.8)	0.81 (0.44, 1.51)	0.513	55 (85.9)	9 (14.1)	1.08 (0.46, 2.52)	0.859
	Non educated	95 (62.5)	57 (37.5)	1		132 (86.8)	20 (13.2)	1	
Occupation	Government employees	15 (57.7)	11 (42.3)	1.47 (0.54, 3.97)	0.450	19 (73.1)	7 (26.9)	1.89 (0.67, 6.34)	0.251
	Self-employed workers	60 (68.2)	28 (31.8)	0.93 (0.43, 2.01)	0.860	68 (77.3)	20 (22.7)	1.60 (0.62, 4.12)	0.329
	Farmers	74(56.9)	56 (43.1)	1.51 (0.74, 3.08)	0.251	117 (90.0)	13 (10.0)	0.60 (0.22, 1.62)	0.313
	Not working	30 (66.7)	15 (33.3)	1		38 (84.4)	7 (15.6)	1	
Monthly household income	≥ 50,000 YER	48 (60.0)	32 (40.0)	1.12 (0.66, 1.90)	0.675	61 (76.2)	19 (23.8)	2.01 (1.05, 3.86)	0.033^a,b^
	< 50,000 YER	131 (62.7)	78 (37.3)	1		181 (86.6)	28 (13.4)	1	
Presence of confirmed CL cases among family members	Yes	40 (75.5)	13 (24.5)	0.47 (0.24, 0.92)	0.025^a,b^	48 (90.6)	5 (9.4)	0.48 (0.18, 1.28)	0.136^b^
	No	139 (58.9)	97 (41.1)	1		194 (82.2)	42 (17.8)	1	
Knowledge about CL	Good	84 (56.8)	64 (43.2)	1.57 (0.97, 2.54)	0.063^b^	121 (81.8)	27 (18.2)	1.35 (0.72, 2.54)	0.350
	Poor	95 (67.4)	46 (32.6)	1		121 (85.8)	20 (14.2)	1	
Knowledge about sandflies	Good	57 (58.2)	41 (41.8)	1.27 (0.77, 2.09)	0.344	79 (80.6)	19 (19.4)	1.40 (0.74, 2.66)	0.302
	Poor	122 (63.9)	69 (36.1)	1		163 (85.3)	28 (14.7)	1	
Attitude towards CL	Positive	–	–	–	–	96 (87.3)	14 (12.7)	0.65 (0.33, 1.27)	0.202^b^
	Negative	–	–	–	–	146 (81.6)	33 (18.4)	1	

*COR* crude odds ratio. *CI* confidence interval.

^a^Significant association (*P* < 0.05).

^b^Included in multivariate analysis (*P* ≤ 0.25).

^c^Based on scores shown in Table 3.

^d^Based on scores shown in Table 4.

The multivariate logistic regression analyses (Table 8) show that females (AOR = 0.53; 95% CI = 0.32, 0.82) and participants working as farmers (AOR = 0.51; 95% CI = 0.33, 0.83) were less likely to have good knowledge about CL compared to their male and non-working counterparts. Participants who lived in the same household with confirmed CL cases were about two times (AOR = 1.94; 95% CI = 1.03, 3.66) more likely to have good CL-related knowledge compared to those in households without confirmed CL cases. Also, the results demonstrate that the odds of having good knowledge about sandflies increased by about two times among participants over 40 years of age compared to younger participants (AOR = 1.95; 95% CI = 1.18, 3.22). Interestingly, participants who lived with confirmed CL cases in the same household were 0.46 times (AOR = 0.46; 95% CI = 0.23, 0.93) less likely to have a good attitude towards CL compared to those living in households without CL cases. Likewise, participants from Razeh were less likely to have a positive attitude towards CL compared to those from Alsomal (AOR = 0.42; 95% CI = 0.23, 0.82). Regarding prevention practices, participants with monthly household incomes of ≥ YER50,000 were more inclined to follow good CL-related prevention practices (AOR = 2.06; 95% CI = 1.07, 3.96) when compared to their peers who had lower incomes. All of the other socio-demographic factors were not retained in the respective multivariate analyses (Table 8).

**Table 8:** Multivariate analysis for predictors of participants’ knowledge, attitude and prevention practices towards cutaneous leishmaniasis.

Variables	AOR	95% CI	P
Good level of knowledge about CL			
Gender (female)	0.53	0.32, 0.82	0.026^a^
Location (Hemiar Alwasat)	2.13	0.87, 5.23	0.097
Location (Razeh)	0.75	0.42, 1.33	0.317
Occupation (farmers)	0.51	0.33, 0.83	0.006^a^
Monthly household income (≥ 50,000 YER)	1.49	0.91, 2.70	0.110
Presence of confirmed CL cases among family members (yes)	1.94	1.03, 3.66	0.040^a^
Good level of knowledge about sandflies			
Age (> 40 years)	1.95	1.18, 3.22	0.012^a^
Location (Hemiar Alwasat)	0.50	0.18, 1.36	0.174
Location (Razeh)	0.65	0.34, 1.23	0.183
Location (Bani Bahr)	0.60	0.35, 1.01	0.054
Presence of confirmed CL cases among family members (yes)	0.57	0.29, 1.14	0.112
Positive attitude towards CL			
Gender (female)	0.50	0.30, 1.03	0.061
Location (Razeh)	0.42	0.23, 0.82	0.012^a^
Presence of confirmed CL cases among family members (yes)	0.46	0.23, 0.93	0.030^a^
Level of knowledge about CL (good)^b^	1.55	0.94, 2.56	0.088
Good level of prevention practices related to CL			
Level of education (secondary & above)	1.71	0.83, 3.55	0.147
Monthly household income (≥ 50,000 YER)	2.06	1.07, 3.96	0.031^a^
Presence of confirmed CL cases among family members (yes)	0.46	0.17, 1.25	0.128
Level of attitude towards CL (positive)^c^	0.580	0.29, 1.16	0.122

*AOR* adjusted odds ratio. *CI* confidence interval.

^a^Significant predictor (*P* < 0.05).

^b^Based on scores shown in Table 1.

^c^Based on scores shown in Table 3.

## Discussion

Although cutaneous leishmaniasis (CL) was perceived as a serious health problem in the study areas, the findings of the present study revealed insufficient knowledge about CL and its sandfly vector, poor attitudes towards prevention and poor CL-related prevention practices. The reported poor levels of knowledge, attitudes and practices (KAP) towards CL could be a direct consequence of the lack of a national control programme for leishmaniasis. In this study, being female and being a farmer were found to be negatively associated with participants’ knowledge about CL. People over the age of 40 years were associated with a good level of knowledge about the sandfly vector. The study also revealed that living in the same household with confirmed CL cases and residing in Razeh sub-district were significant factors associated with a negative attitude towards CL prevention. Household monthly income was the significant factor associated with CL prevention practices. Information about people’s KAP concerning this neglected disease and its vector in endemic areas is imperative for developing effective preventive and control interventions and promoting community mobilisation to achieve and sustain disease elimination.

Although all the participants had prior knowledge about the disease by its local name and about two-thirds of them knew it by the scientific term ‘*Leishmania’*, the overall level of knowledge about the disease and its sandfly vector was low. Only 9.3% of the participants had correct knowledge about the role of sandflies in CL transmission, while about one third of them were able to identify and differentiate sandflies from other mosquitoes and house flies. The only previous KAP local survey conducted in Taiz Governorate, southwestern Yemen reported that only 22.3% of the participants had a good level of knowledge about CL^27^, which is much lower than that reported by the present study. It is possible that the higher awareness about CL’s name and its clinical presentation can be linked to the hyperendemicity of the disease in the Utmah district^28^. Indeed, the majority of the studied participants had seen CL cases among household members or other individuals within the community. While both studies revealed a significant association between individuals over the age of 40 years and a good level of knowledge on CL, it is notable that more than half of the studied participants from the Utmah district were aged over 40 years, whereas about one-third of those from Taiz were in this age group^27^.

In comparison with KAP surveys conducted in CL-endemic areas in other countries, a previous study in rural communities of southern Ethiopia found that all heads of households studied did not know the mode of CL transmission and had never heard of sandflies, with only 19% (80/422) having a good level of CL-related knowledge^18^. Similarly, very poor levels of knowledge about the disease and its vector were reported in endemic areas of Kerala, India^19^ and Khyber Pakhtunkhwa, Pakistan^14^. On the other hand, a previous study in central Iran demonstrated that almost all (97.9%) participants were aware that CL is transmitted by sandflies; however, only 28.6% were able to identify a sandfly^15^. The much higher level of knowledge on sandflies as the vector compared to our study could be due to differences in study population and settings. The Iranian study was conducted among middle- and high-school students who were the most at-risk population in the study area and most of them received knowledge about the disease from healthcare personnel^15^.

The present study demonstrated that majority of the participants had a negative attitude towards CL prevention and this finding is consistent with similar studies conducted in Yemen and Saudi Arabia^16,27^. On the contrary, the majority of participants in northern Ethiopia (82%) had positive attitudes about the prevention of CL^35^. The endemicity of CL for many decades, coupled with the lack of health education interventions, may result in negative attitudes regarding the possibility of CL prevention. Interestingly, the present study revealed that nearly half of the participants, particularly females, believed that CL is a stigmatising disease. This concurs with a previous study conducted among CL-infected females in Sana’a and Radaa governorates^36^. In the present study, most CL lesions were on the face, particularly on the cheeks, nose and lips^28^, and the infected female participants have expressed their strong sense of shame and embarrassment of being seen in public. CL-associated stigmatisation might prevent infected individuals, particularly females, from seeking treatment at health centres^37,38^. The stigma associated with CL can result in social discrimination, isolation and rejection, and these may also exacerbate their health outcomes and influence their educational attainment^36,39,40^.

Poverty prevails in the studied communities and this is in line with the fact that over 80% of poor people in Yemen reside in rural areas^41^. Thus, poor housing and environmental conditions that favour CL transmission represent critical challenges that may hinder the control and elimination of the disease in Utmah. Poor housing conditions like houses with mud-plastered stone walls or wooden roofs and presence of cracks and crevices in house walls provide suitable resting and hiding places for sandflies after feeding and these might be associated with a higher risk of exposure to vector bites and CL infection^42,43^. The findings showed that the majority of the population did not adopt any control measure, with a very small proportion of the households owning bed nets and/or using insecticides. Consistent with our findings, previous studies conducted in CL-endemic rural communities of Ethiopia and Pakistan found negligible proportions of the participants used bed nets and insecticides, and the authors have attributed this to the participants’ poor level of knowledge about sandflies and preventive measures^14,18^. Therefore, health education intervention about the vector and prevention of CL is crucial for the endemic areas to adopt necessary preventive and control measures^44^.

In regard to treatment-seeking behaviour, over half of the studied participants declared that they would go to the nearest health centre as a first line activity to seek treatment for skin lesions; however, in reality, the majority of the participants used traditional remedies to treat previous CL-suspected lesions. These findings are consistent with previous studies in Yemen^27^ and southern Ethiopia^18^. Other studies from northern Ethiopia^35^ and India^19^ found that 90% and 100% of participants with CL, respectively, were treated solely by traditional herbal remedies. Consistent with our findings, previous studies conducted in different countries such as Ethiopia^18^, Iran, and Ecuador^45^ showed that cauterisation by placing very hot metal objects or applying battery acid on the lesions is one of the common traditional methods used to treat CL. Awareness of CL treatments should be improved, and adequate chemotherapy should be made available and accessible in endemic areas.

In the present study, the knowledge about CL was found to be associated with sex, occupation and the presence of CL-confirmed cases in the household. The female participants and those working as farmers were less likely to have good CL-related knowledge compared to their counterparts. Similar findings were reported in Yemen and elsewhere^18,27^. Females also had lower exposure to CL-related knowledge at health centres or from health workers visiting the villages, potentially due to the gender inequality in preferential treatment and the cultural customs that do not allow women to deal with stranger men including health workers^31,46^. Our findings were the same as those in the previous study from Yemen that participants aged over 40 years old had better knowledge on sandflies compared to younger participants^27^. In CL-endemic areas, it is expected that elder people gain knowledge about the disease and sandflies over time. In addition, this study found that participants who lived with confirmed CL cases in the same household and those from the Razeh sub-district had significantly lower odds of having a positive attitude towards CL. Although the prevalence of CL-confirmed cases across the studied sub-districts was comparable^28^, the observed district-wise difference in the attitude towards CL could be due to participants’ experience with the disease. The monthly household income was retained as a significant predictor of participants’ CL-related prevention practices. Indeed, CL is considered to be a poverty-related NTD^47^. Poverty, represented by low income and limited financial resources, has adverse effects on the prevention-related practices of vector-borne diseases by limiting the opportunities for families to improve housing conditions or to adopt protective measures, such as bed nets and insecticides or repellents^48^.

It is important to acknowledge that the cross-sectional study design used in the study limited our ability to infer causal relationship between participants’ KAP and the identified significant predictors. Rural communities in the western highland of Yemen have almost similar socioeconomic, environmental, and health profiles; thus, the findings of the present study might be generalisable to other CL-endemic rural areas in this part of the country.

## Conclusion

This study revealed that the overall knowledge, attitude and prevention practices towards CL and its sandfly vector among the study population were poor. The majority of the participants had a poor understanding regarding the appropriate treatment of CL, opting instead for traditional methods to manage their skin lesions. Age, sex, occupation, presence of CL-confirmed cases in the same household, and household monthly income were the significant predictors associated with KAP towards CL among the participants. These findings emphasize that health education campaigns concerning CL transmission, preventive measures, and appropriate treatment should be offered to this population, with special attention given to females, individuals aged below 40 years old, and farmers. Also, providing treatment and protective materials like bed nets and insecticide spraying, particularly for households with low income and limited financial resources should be considered. Indeed, this can be integrated with the national malaria control programme. In such fragile and conflict-affected settings, the provision of mobile health clinics targeting CL and other infectious diseases could be a proper approach to maximising vulnerable population coverage.

### Supplementary Information

Supplementary Information.

## Data Availability

All data generated or analysed during this study are included in this published article and its supplementary information files.
