# Supplementary material for: Knowledge, attitude, practices and treatment-seeking behaviour concerning cutaneous leishmaniasis among rural hyperendemic communities in western Yemen
Source: Sci Rep. 2024 Jun 3;14:12662. doi: 10.1038/s41598-024-63526-6 (PMC11148075; doi:10.1038/s41598-024-63526-6)
Supplement: Supplementary file 1 — Supplementary Information. [file 41598_2024_63526_MOESM1_ESM.docx]

**Supplementary Table S1.** General demographic and socioeconomic characteristics of the household heads participated in the survey (n = 289)

| Variables |  | Total  N (%) |
| --- | --- | --- |
| Age (years) | 18–30 | 66 (22.8) |
|  | 31–40 | 75 (26.0) |
|  | 41–50 | 65 (22.5) |
|  | > 50 | 83 (28.7) |
| Gender | Female | 72 (24.9) |
|  | Male | 217 (75.1) |
| Location | Hemiar Alwasat | 24 (8.3) |
|  | Bani Bahr | 111 (38.4) |
|  | Razeh | 69 (23.9) |
|  | Alsomal | 85 (29.4) |
| Level of education | Non-educated | 152 (52.6) |
|  | Primary | 64 (22.1) |
|  | Secondary & above | 73 (25.3) |
| Occupation | Government employees | 26 (9.0) |
|  | Self-employed workers | 88 (30.4) |
|  | Farmers | 130 (45.0) |
|  | Unemployed | 45 (15.6) |
| No. of household members | > 10 | 125 (43.3) |
|  | 6–10 | 116 (40.1) |
|  | ≤ 5 | 48 (16.6) |
| Monthly household income | ≥ 50,000 YER | 80 (27.7) |
|  | < 50,000 YER | 209 (72.3) |
| Housing condition | Good (brick, concrete or stone, no cracks) | 53 (18.3) |
|  | Poor (mud cracked walls) | 236 (81.7) |
| Sources of drinking water | Improved (piped) | 18 (6.2) |
|  | Unimproved (rain, well, springs) | 271 (93.8) |

YER, Yemeni Riyal; (US$1 = YER650).
